# Natural-cause mortality and C-reactive protein levels in patients with schizophrenia spectrum disorders: A prospective total cohort study

**DOI:** 10.1016/j.bbih.2025.101133

**Published:** 2025-11-04

**Authors:** F. Fathian, I. Divkovic, M. Fagerbakke Strømme, A. Mykletun, R.A. Kroken, C.A. Bartz-Johannessen, E. Johnsen

**Affiliations:** a Division of Psychiatry, Haukeland University Hospital, Bergen, Norway; b Mohn Research Centre for Psychotic Disorders, Haukeland University Hospital, Bergen, Norway; c Department of Clinical Medicine, University of Bergen, Bergen, Norway; d Norwegian Institute of Public Health, Oslo, Norway; e University of Tromsø, Tromsø, Norway; f Nordland Hospital, Bodø, Norway

**Keywords:** C-reactive protein, Schizophrenia, Mortality

## Abstract

**Background:**

Schizophrenia spectrum disorders (SSD) are associated with an excess mortality risk compared with the general population. The involvement of low-grade inflammation and elevated C-reactive protein (CRP) levels is well established in SSD. However, associations between CRP and mortality risk in SSD are less investigated.

**Aim:**

To investigate the association between the baseline CRP level and natural-cause mortality risk in SSD.

**Methods:**

We included all patients with an SSD diagnosis and baseline CRP measurement from an open cohort study of consecutively admitted patients to a psychiatric acute unit at Haukeland University Hospital, Bergen, Norway, between May 1, 2005 and June 15, 2014. All patients were followed until the time of death or censoring/study end, up to 18.6 years, and only the assessments at admission were the focus of the present study. A competing risk model was used, adjusting for age and sex.

**Results:**

Among 1315 individuals, 245 (19 %) died of natural causes; among these patients, 66 (27 %) deaths were related to cardiovascular disease (CVD). Elevated baseline CRP levels of 1.0 ≤ CRP <3.0 mg/L were significantly associated with increased natural-cause mortality risk (adjusted hazard ratio [AHR], 1.88; 95 % confidence interval [CI], 1.22–2.88; p-value, 0.004), with a stronger association for CRP ≥3.0 mg/L (AHR, 2.28; 95 % CI, 1.50–3.48; p-value, <0.001), compared with patients with CRP <1.0 mg/L. Moreover, baseline CRP ≥3.0 mg/L was associated with increased CVD-related mortality risk (AHR, 3.12; 95 % CI, 1.16–8.41; p-value, 0.024).

**Conclusions:**

Elevated CRP levels were associated with increased natural-cause mortality and, specifically, with CVD-related mortality risk. The CRP level may thus be considered a predictive factor in mortality risk scoring algorithms in SSD.

## Highlights

• C- reactive protein (CRP) was associated with mortality in schizophrenia.
• Increased natural-cause mortality was associated with CRP ≥1.0 mg/L.
• Cardiovascular mortality counted for 27 % of natural-cause death.
• C-reactive protein may be considered as a mortality risk predictor.

## 1 Introduction

Schizophrenia is a severe mental disorder characterised by a complex array of psychotic, cognitive, mood, and motor symptoms, affecting approximately 1 % of the population (Jauhar et al., 2022). In addition to the burden of symptoms, patients with schizophrenia have a 2.5-fold increase in age-adjusted mortality compared with the general population (Hjorthøj et al., 2017). A study of the Danish population found a reduced life expectancy of 13.5 years in men and 11.4 years in women with schizophrenia, respectively (Laursen et al., 2019). A large systematic review and meta-analysis of 135 studies, including 4.5 million patients with schizophrenia (Correll et al., 2022), identified specific causes of unnatural death with the highest mortality risk (risk ratio [RR]) as suicide, injury–poisoning, or undetermined non-natural causes (RR = 9.76–8.42). These were followed by natural causes of death, including pneumonia (RR = 7.00); other infections and endocrine, respiratory, urogenital, or diabetes-related causes (RR = 3–4); alcohol-related, gastrointestinal, renal, nervous system-related, cardio-cerebrovascular, or all-natural causes (RR = 2–3); and liver-related, cerebrovascular, or any cancer-related causes (RR = 1.33–1.96) (Correll et al., 2022). Given the high prevalence of cardiovascular disease (CVD) as a cause of death in the general population (Ahmad et al., 2024), increased CVD risk is numerically one of the primary contributors to elevated mortality in schizophrenia (Correll et al., 2022; Olfson et al., 2015). Patients with schizophrenia exhibit high rates of CVD risk factors, including metabolic syndrome and diabetes, as well as risk behaviours such as physical inactivity, smoking, and alcohol and illicit drug use. These factors also contribute to poorer outcomes, such as increased COVID-19 severity and mortality, compounded by complex genetic underpinnings and lower adherence to recommendations for healthy lifestyle habits (Correll et al., 2022; Firth et al., 2020; Tzur Bitan et al., 2021; Vancampfort et al., 2017). Finally, studies have shown a higher mortality rate in patients with schizophrenia during periods when they are not using antipsychotic medication than during periods when they are receiving such treatment (Strømme et al., 2021; Tiihonen et al., 2006).

From a pathophysiological perspective of cardiovascular events, the role of inflammation in the atherosclerotic disease process is well established (Ross, 1999). The development of atherosclerotic plaque begins with an inflammatory response to injury triggered by atherogenic factors—i.e., smoking, hypertension, dyslipidaemia, and hyperglycaemia (Libby and Ridker, 1999; Pearson et al., 2003; Plutzky, 2001). Inflammation plays a role in all stages of atherothrombosis, which is the underlying cause of approximately 80 % of all sudden cardiac deaths (Albert et al., 2002).

The acute-phase protein C-reactive protein (CRP) is one of the most sensitive biomarkers of inflammation. It is synthesised in the liver, particularly in response to interleukin-6 (IL-6) or tumour necrosis factor-α (Castell et al., 1990; Mantovani and Garlanda, 2023). Elevated CRP levels are observed in response to infection, inflammatory conditions, trauma, and ageing (Pepys and Hirschfield, 2003). Interestingly, many established cardiovascular risk factors—such as diabetes, smoking, dyslipidaemia, and hypertension—are associated with increased CRP levels (Lee et al., 2009; Schillaci and Pirro, 2006; Sesso et al., 2003; Tracy et al., 1997; Verhagen et al., 2013). Furthermore, elevated CRP levels are associated with the risk of other conditions such as cancer and chronic obstructive pulmonary disease (Guo et al., 2013; Il'yasova et al., 2005; Siemes et al., 2006; van Durme et al., 2009), all of which are among the main natural causes of mortality in individuals with schizophrenia spectrum disorders (SSD) (Correll et al., 2022). CRP and its mechanism of action are thought to be linked to endothelial dysfunction. CRP binds to low-density lipoprotein (LDL) and is detected in atherosclerotic plaques (Koenig, 2013; Pepys et al., 1985; Zhang et al., 1999). This reliable and widely available inflammatory marker is used in the monitoring of various disease activities, and as a marker of atherosclerosis in cardiovascular risk stratification and monitoring of CVD-related events (Deodhar, 1989; Du Clos, 2000; Pepys and Hirschfield, 2003; Ridker, 2016). The American Heart Association and Centers for Disease Control and Prevention classify a CRP concentration of >3.0 mg/L as indicating a high risk of CVD (Pearson et al., 2003). Elevated CRP levels can independently predict the risk of all-cause and CVD mortality in the general population (Li et al., 2017; Proctor et al., 2015). Interestingly, Hageman et al. (2022) presented an updated Secondary Manifestations of ARTerial disease (SMART2) risk score—a validated tool for predicting recurrent atherosclerotic CVD—based on their 10-year risk assessment study, which includes CRP in its algorithm (Hageman et al., 2022).

Furthermore, a collaborative study of three multinational randomised trials involving statin-treated patients with, or at risk of, atherosclerotic disease showed that a CRP level of ≥2.0 mg/L was a stronger predictor of future major adverse CVD events, CVD mortality, and all-cause mortality than was a LDL cholesterol (LDL-C) level of ≥70.0 mg/dL (Paul M. Ridker et al., 2023a, Ridker et al., 2023b). Several studies have, however, identified potential confounders—such as body mass index, age, sex, smoking, alcohol, and illicit drug use—that must be accounted for in the analysis of CRP levels (Kirkpatrick and Miller, 2013).

Inflammation, with increased levels of immune markers including CRP, is well established in schizophrenia (Fernandes et al., 2016; Fond et al., 2018; Halstead et al., 2023; Miller et al., 2014; Proctor et al., 2015; Wang et al., 2017; Yuan et al., 2019). According to a meta-analysis of 50 studies involving patients with schizophrenia, CRP levels were higher than in controls, with a further increase during acute phases (Lestra et al., 2022). Increased CRP levels are associated with more severe symptomatology (Dal Santo et al., 2024; Johnsen et al., 2016; Patlola et al., 2023; Steiner et al., 2020), treatment resistance (Llorca-Bofí et al., 2024), and cardiovascular comorbidity (Xie et al., 2023). Furthermore, elevated CRP levels in patients with schizophrenia may correlate with increased all-cause mortality (Horsdal et al., 2017; Llorca-Bofí et al., 2024). Findings from a Danish 10-year follow-up registry-based study showed that elevated baseline levels of CRP, leucocytes, neutrophils, monocytes, and the leucocyte-, neutrophil-, and monocyte-to-lymphocyte count ratios were associated with increased age-adjusted mortality risk. Interestingly, CRP emerged as the strongest predictor of age-adjusted mortality (Llorca-Bofí et al., 2024). However, the association was only observed for CRP levels of >10.0 mg/L, which is well above the American Heart Association and Centers for Disease Control and Prevention's high-risk threshold of >3.0 mg/L (Pearson et al., 2003).

To summarise, the role of CRP as one of the essential tools for CVD risk assessment in internal medicine and cardiology is well established, and there is growing evidence supporting a more proactive approach to lipid-lowering treatment, including combination with inflammation-inhibiting therapy (Xie et al., 2024; Xue et al., 2024). However, the value of CRP level measurement as a means of ultimately reducing mortality risk in patients with schizophrenia remains to be determined. To this end, new large-scale prospective studies involving individuals with schizophrenia are needed. Accordingly, our primary aim was to investigate the association between CRP levels and subsequent natural-cause mortality in individuals with SSD through a total-cohort study of patients consecutively admitted to the psychiatric acute unit within a large catchment area. The secondary aim was to examine associations between CRP levels and CVD-specific mortality rates.

## 2 Material and methods

### 2.1 Study design

This study was based on data collected as part of the Suicidality in Psychiatric Emergency Admissions (SIPEA) study (Mellesdal et al., 2010) and its continuation, the SIPEA-II study. The material and methods have been described in greater detail in a previous publication (Strømme et al., 2021). All patients admitted to the 19-bed psychiatric acute unit at the Division of Psychiatry, Haukeland University Hospital in Bergen, Norway, between May 1, 2005 and June 15, 2014 were included in the SIPEA and SIPEA-II studies. Data were obtained through mapping of patient medical records at admission and included tobacco smoking status, blood pressure, body mass index (BMI), CRP, LDL-C and glycosylated haemoglobin (HbA1c). All patients were followed until the time of death or censoring/study end, up to 18.6 years, and only the assessments at admission were the focus of the present study. Follow-up data were retrieved from the Norwegian Cause of Death Registry for the period 2005–2023. The hospital's catchment area comprises approximately 400,000 inhabitants, and around 95 % of all acute psychiatric admissions from this area during the study period were directed to this unit. Clinicians who assessed patients at admission underwent training in the use of the rating scales applied. The inclusion criteria for the present study were having a diagnosis of SSD (F20–F29) according to the International Classification of Diseases, Tenth Revision (https://icd.who.int/browse10/2019/en), and a registered CRP value at baseline. CRP measurements had to be recorded either during or after the same treatment period in which the SSD diagnosis was made (i.e., CRP values measured prior to the diagnosis were excluded from the analyses). There were no exclusion criteria for this study. CRP was measured using the Tina-quant C-reactive Protein (Latex) assay on the Roche Modular P® system, which detects CRP levels of >1.0 mg/L. LDL-C was measured using the enzymatic colorimetric test on the Roche modular P, with a reference range of 1.8–5.7 mmol/L. HbA1c was measured using High performance liquid chromatography (HPLC) on the Biorad Variant II, with a reference range of 4.0–6.4 % for non-diabetic patients. Information on mortality was obtained by linking patients' national identity numbers to data from the Norwegian Cause of Death Registry.

The study complies with the ethical standards of the relevant national and institutional committees on human experimentation and with the Helsinki Declaration of 1975, as revised in 2008. It was approved by the Norwegian Social Science Data Service, the Norwegian Directorate of Health, and the Regional Committee for Medical Research Ethics (approval number REK 46004). Use of patient information without informed consent was authorized by the abovementioned instances.

### 2.2 Statistical analyses

Statistical analyses were conducted in R (Team, 2020). For the primary analysis, the time from CRP measurement to the occurrence of natural death, death of other causes, censoring, or study end was used in a competing risk model to investigate the association between baseline CRP level and the risk of natural death. Patients were divided into three groups based on their CRP level: CRP <1.0 mg/L, 1.0 ≤ CRP <3.0 mg/L, and CRP ≥3.0 mg/L. The CRP variable, along with age and sex, was included as an explanatory variable in the competing risk model for the primary analysis. The same model was used in the secondary analysis, which included only deaths due to CVD.

A sensitivity analysis was conducted using the same dataset, in which the following potential confounders were included in addition to the explanatory variables in the primary model: BMI, tobacco smoking status (yes/no), blood pressure, LDL-C, and HbA1C. These additional confounders were included only in the sensitivity analyses due to substantial missing data. Multiple imputation was performed to address the missing data, and a Cox regression model was fitted to the imputed dataset. The level of statistical significance was set at α = 0.05, two-sided.

## 3 Results

In total, 1315 patients with a SSD diagnosis and an available CRP measurement, as defined by the study design, were included between May 1, 2005 and June 15, 2014. Their baseline mean age was 42.5 years, and 44.2 % were female. A total of 32.2 % had schizophrenia and 5.1 % had comorbid alcohol or substance use disorder. Furthermore, 35.4 % of 760 available blood pressure fulfilled criteria for hypertension. Baseline mean values for BMI was 26.2; LDL-C was 3.0 mmol/L; and HbA1c was 5.6 %. A total of 349 patients (26.5 %) had CRP levels <1.0 mg/L, whereas 490 patients (37.3 %) had CRP levels 1.0 ≤ CRP <3.0 mg/L; and for 476 patients (36.2 %), CRP levels were ≥3.0 mg/L. Additional baseline characteristics are presented in Table 1.

**Table 1:** Baseline characteristics of the study sample (F20–F29) at first admission with CRP measurement (n = 1315)^a^.

Variable	Mean	SD
Age at inclusion, years	42.5	16.1

Sex	**n**	**Percent**
Male	734	55.8
Female	581	44.2
Tobacco smoking^a^ (n = 820)	447	54.5

F20–F29		
F20 Schizophrenia	424	32.2
F21 Schizotypal disorder	16	1.2
F22 Delusional disorders	343	26.1
F23 Brief psychotic disorder	263	20.0

F25 Schizoaffective disorders	129	9.8
F24 Shared psychotic disorder and F28 Other psychotic disorder	23	1.7
F29 Unspecified psychosis	117	8.9
F10.0–F19.9 Comorbid alcohol or substance use disorder		
Yes	67	5.1
No	1248	94.9
Hypertension^b^ (n = 760)	269	35.4

	**Mean/Median**	**SD**
BMI^a^, kg/m^2^ (n = 486)	26.2	5.9
LDL-C^a^, mmol/L (n = 614)	3.0	1.1
HbA1c^a^, % (n = 592)	5.6	1.0
CRP, mg/L	6.1/2.0	14.6

Notes:

a Substantial proportion with missing data.

b Hypertension defined as systolic blood pressure of ≥140 mmHg or/and diastolic blood pressure of ≥90 mmHg; SD = standard deviation; F = ICD-10 diagnosis at discharge; CRP = C-reactive protein; BMI = body mass index; LDL-C = low-density lipoprotein cholesterol; HbA1c = glycosylated haemoglobin.

During the 18.6-year follow-up period, a total of 342 patients died, of whom 245 (26 %) were classified as having died of natural causes. The mean age of natural death with standard deviation (SD) was 68.5 (SD 14.4) years. Among the natural deaths, a total of 66 (27 %) were due to CVD, a total of 58 (24 %) to respiratory system diseases, a total of 55 (22 %) to neoplasms, a total of 8 (3 %) to infections, two (1 %) to diabetes mellitus, and finally 56 (23 %) to other conditions.

The results from our primary analysis—both multivariate and univariate—examining the association between baseline CRP level and natural-cause death are presented in Table 2. Baseline levels of 1.0 ≤ CRP <3.0 mg/L were associated with an 88 % increased risk of natural-cause mortality, and baseline CRP ≥3.0 mg/L was associated with a 128 % increased risk, compared with patients with CRP <1.0 mg/L.

**Table 2:** Univariate and multivariate analyses of association between baseline CRP level and natural-cause mortality.

Variable	Multivariate analysis			Univariate analysis		
	AHR	95 % CI	p-value	HR	95 % CI	p-value
1.0 ≤ CRP <3.0 mg/L	1.88	1.22, 2.88	0.004	2.96	1.90, 4.63	<0.001
CRP ≥3.0 mg/L	2.28	1.50, 3.48	<0.001	4.06	2.62, 6.29	<0.001
Age	1.09	1.08, 1.10	<0.001	1.09	1.08, 1.10	<0.001
Female sex	0.70	0.53, 0.91	0.009	1.39	1.09, 1.79	0.009

**Notes:** AHR = adjusted hazard ratio; HR = hazard ratio; Reference group is CRP <1.0 mg/L; CI = confidence interval; CRP = C-reactive protein.

The results from the secondary analysis, showing the association between baseline CRP level and CVD mortality, are presented in Table 3. The risk of cardiovascular mortality was increased 212 % for baseline CRP ≥3.0 mg/L compared with patients with CRP <1.0 mg/L.

**Table 3:** Association between baseline CRP level and cardiovascular mortality.

Variable	AHR	95 % CI	p-value
1.0 ≤ CRP <3.0 mg/L	2.08	0.77, 5.62	0.15
CRP ≥3.0 mg/L	3.12	1.16, 8.41	0.024
Age	1.06	1.05, 1.07	<0.001
Female sex	0.85	0.51, 1.41	0.53

**Notes:** AHR = adjusted hazard ratio; Reference group is CRP <1.0 mg/L; CI = confidence interval; CRP = C-reactive protein.

Finally, the sensitivity analysis, which included additional confounders (BMI, tobacco smoking, blood pressure, LDL-C, and HbA1c showed that baseline levels of 1.0 ≤ CRP <3.0 mg/L were associated with 87 % increased risk of natural-cause mortality, and baseline CRP ≥3.0 mg/L associated with 113 % increased risk compared with CRP <1.0 mg/L (Table 4).

**Table 4:** Adjusted analysis of association between baseline CRP level and natural-cause mortality.

Variable	AHR	95 % CI	p-value
1.0 ≤ CRP <3.0 mg/L	1.87	1.16, 3.02	0.009
CRP ≥3.0 mg/L	2.13	1.32, 3.45	0.002
Age, years	1.10	1.09, 1.11	<0.001
Female sex	0.71	0.53, 0.97	0.027
Tobacco smoking	1.63	1.08, 2.45	0.019
BMI, kg/m^2^	0.99	0.95, 1.04	0.700
Hypertension	0.77	0.52, 1.14	0.191
LDL-C, mmol/L	0.85	0.72, 1.01	0.057
HbA1c, %	1.06	0.90, 1.25	0.483

**Notes:** AHR = adjusted hazard ratio; Reference group is CRP <1.0 mg/L; CI = confidence interval; CRP = C-reactive protein; BMI = body mass index; Hypertension = defined as systolic blood pressure of ≥140 mmHg and/or diastolic blood pressure of ≥90 mmHg; LDL = low-density lipoprotein cholesterol; HbA1c = glycosylated haemoglobin.

## 4 Discussion

The main findings of this cohort study of 1315 patients were the associations between increased baseline CRP levels and mortality risk in individuals with SSD. Baseline levels of 1.0 ≤ CRP <3.0 mg/L were associated with an 88 % increased risk of natural-cause mortality, and baseline CRP ≥3.0 mg/L was associated with a 128 % increased risk, compared with patients with CRP <1.0 mg/L. These associations remained significant after adjusting for additional confounders. Furthermore, similar but stronger associations between baseline CRP levels ≥3 mg/L and cardiovascular-related mortality risk were observed in the secondary analyses. The novelty of our findings is the impact of lower CRP levels being associated with natural-cause mortality, which is even lower than the threshold of the American Heart Association and Centers for Disease Control and Prevention, which classifies CRP concentration of >3.0 mg/L as high risk of CVD (Pearson et al., 2003). Another population study in people with SSD also found increased all-cause mortality but only for baseline CRP levels >10 mg/L (Llorca-Bofí et al., 2024). Their study sample was on average 26 years younger than our sample, which together with a shorter follow-up time of 10 years may explain some of the discrepancy towards our findings. The predictive value of CRP levels for mortality in patients with SSD has been less frequently investigated, and findings are mixed. Most evidence comes from population- or registry-based studies showing associations between elevated CRP levels and mortality in SSD (Horsdal et al., 2017; Llorca-Bofí et al., 2024). A systematic review and meta-analysis of mortality risk in people with schizophrenia showed that individuals with first-episode and incident schizophrenia faced the highest mortality risk compared with the general population (Correll et al., 2022). Patients with first-episode psychosis have shown increased levels of inflammatory markers, particularly tumour necrosis factor-α and CRP, especially patients exposed to early life adversity (Di Nicola et al., 2013; Hepgul et al., 2012). This suggests the presence of a low-grade inflammatory state from early adulthood, which may contribute to elevated CVD risk over time. Such a mechanism could help explain the increased mortality risk observed even within the CRP 1.0–3.0 mg/L range in patients with SSD. Thus, reducing inflammation from the early stages may be warranted.

In our sensitivity analysis, age, female sex, and tobacco smoking were associated with a modest influence on natural-cause mortality risk. Overall, our findings suggest that an elevated CRP level of ≥1.0 mg/L independently predicts natural-cause mortality. Findings from aforementioned Danish study, which included measurements of white blood cell counts and/or CRP at the first diagnosis of SSD, shed light on the immunological processes in SSD (Llorca-Bofí et al., 2024). Elevated baseline CRP levels of >10.0 mg/L, leucocytes, neutrophils, monocytes, and leucocyte-, neutrophil-, and monocyte-to-lymphocyte count ratios were associated with increased mortality risk, with CRP emerging as the strongest predictor. CRP levels of >10.0 mg/L are often considered indicative of acute inflammatory processes such as infections, inflammation, or other non-CVD conditions (Pearson et al., 2003). However, in line with other studies, we included higher CRP values in our analyses because several investigations have shown independent associations between CRP levels of >10.0 mg/L and both recurrent CVD and all-cause mortality, with relative risks exceeding those associated with CRP levels of ≤10.0 mg/L (Burger et al., 2023; Carrero et al., 2019; Sabatine et al., 2007).

Interestingly, Xie et al. (2023), using the Framingham cardiovascular risk score (Wilson et al., 1998) in patients with schizophrenia, found that the high-risk group had higher CRP levels than the non-high-risk group and that a CRP level of ≥2.13 mg/L was significantly associated with increased CVD risk in this population (Xie et al., 2023). It is therefore essential to investigate whether clinically available markers such as CRP can serve as valid predictors of mortality risk in SSD.

The mechanism of action by which CRP correlates with mortality risk have been shown to be linked to endothelial dysfunction leading to atherosclerosis, CVD-events and mortality (Koenig, 2013; Pearson et al., 2003). The well-replicated finding of elevated CRP levels in this patient group compared with the general population (Lestra et al., 2022) likely reflects a long-lasting inflammatory process leading to multimorbidity and increased mortality risk.

Moreover, identifying safe and effective CRP-lowering treatment strategies to reduce CVD risk and events is of critical importance. Ridker et al. (1997) were among the first to demonstrate the predictive role of baseline CRP levels for future myocardial infarction (MI) and stroke and using CRP lowering anti-inflammatory agents as CVD-preventive agents. Findings from the Scandinavian Simvastatin Survival Study Group (4S) study of patients with coronary heart disease have been a landmark in demonstrating the efficacy of statin therapy in reducing recurrent MI, stroke, and CVD-related death as secondary prevention for overt hyperlipidaemia since 1994 (Pedersen et al., 2004). Statins also exert anti-inflammatory effects and according to a systematic review and meta-analysis of patients with CVD, statins reduce CRP levels (Kandelouei et al., 2022). Interestingly, findings from a collaborative analysis of 31,245 patients receiving statins, with or at high risk of atherosclerotic disease, revealed that CRP was a stronger predictor of future cardiovascular events and death than LDL-C, underscoring the importance of combining lipid-lowering and inflammation-inhibiting therapies for optimal CVD risk reduction (Ridker et al., 2023a, Ridker et al., 2023b). Moreover, there are other therapeutic interventions that show anti-inflammatory and CRP-reducing effects. In the CANTOS study of patients with a history of MI and a CRP level of ≥2.0 mg/L, canakinumab a monoclonal antibody targeting IL-1β, lowered CRP levels without affecting LDL-C and resulted in a reduced incidence of recurrent CVD events compared with placebo (Ridker et al., 2017). Similarly, in the RESCUE trial, individuals at high atherosclerotic risk but without any clinically apparent inflammatory conditions, the IL-6 ligand monoclonal antibody ziltivekimab markedly reduced multiple biomarkers of systemic inflammation associated with the atherothrombotic process (Ridker et al., 2021).

Altogether, increasing evidence confirms the effect of CRP lowering agents with specified mechanisms of action on CVD event reduction, however, these findings need to be replicated in patients with SSDs as well.

Some limitations to our study need mentioning. One is the sample including only acutely admitted patients with SSD to an acute psychiatric ward, which have implications on generalisability of the results. The sample accordingly represent the most severely ill subgroup of patients with SSD and may not be representative of out-patients without hospital admissions or those with more stable courses of illness. Furthermore, absence of a healthy control group restricts any comparison to the general population, and lack of leucocyte counts and other inflammatory markers limit inference to inflammation per se. Substantial proportions with missing values for some of the confounding variables increases the risk of selection bias. Therefore, these confounders were only included in sensitivity analyses. With regard to the specific association between CRP levels and cardiovascular mortality, additional confounder adjustment was not performed because of the limited sample size when analysing CVD-related deaths alone. The possibility of residual confounding cannot be ruled out with certainty, but we do consider our results to be robust as the key confounding factors according to previous literature have been adjusted for.

A key strength of the study is its large, longitudinal, unselected total cohort design from a single site, with up to 18.6 years of follow-up and inclusion of all acutely ill patients with SSD. Our sample is therefore highly representative of patients with schizophrenia admitted to a psychiatric acute unit and is comparable to nationwide register studies with larger sample sizes. To our knowledge, this is one of the few prospective longitudinal studies investigating whether CRP levels can predict mortality risk in SSD.

To summarise, findings from our study suggest that a CRP level of ≥1.0 mg/L may be an independent predictor of increased risk of natural-cause mortality and that a CRP level of ≥3.0 mg/L may be associated with increased cardiovascular risk in patients with SSD. Further prospective studies are needed to determine the CRP threshold for evaluating cardiovascular risk in schizophrenia. Our findings support the potential role of baseline CRP measurement in cardiovascular risk stratification, alongside other risk factors, to help reduce excess natural-cause mortality in this already highly burdened population. It is also essential to explore new anti-inflammatory and lipid-lowering strategies to reduce CVD risk and mortality in patients with SSD.

## CRediT authorship contribution statement

**F. Fathian:** Writing – review & editing, Writing – original draft, Supervision, Methodology, Investigation, Conceptualization. **I. Divkovic:** Writing – review & editing, Conceptualization. **M. Fagerbakke Strømme:** Writing – review & editing. **A. Mykletun:** Writing – review & editing, Conceptualization. **R.A. Kroken:** Writing – review & editing. **C.A. Bartz-Johannessen:** Writing – review & editing, Visualization, Software, Formal analysis, Data curation. **E. Johnsen:** Writing – review & editing, Writing – original draft, Validation, Supervision, Resources, Project administration, Methodology, Investigation, Data curation, Conceptualization.

## Data sharing statement

The data collected are not publicly available because of a lack of approval from the regulatory authorities in Norway.

## Funding

The study was funded by the Division of Psychiatry of 10.13039/501100010586Haukeland University Hospital and received additional support through research grants from the Western Norway Regional Health Authority (Grant numbers: 911209/HV and 911671/HV). The funders were not involved in the design or conduct of the study; the collection, management, analysis, or interpretation of the data; the preparation, review, or approval of the manuscript; or the decision to submit the manuscript for publication.

## Declaration of competing interest

The authors declare that they have no known competing financial interests or personal relationships that could have appeared to influence the work reported in this paper.

## Data Availability

The authors do not have permission to share data.
